# Time-Varying Environmental and Polygenic Predictors of Substance Use Initiation in Youth: A Survival and Causal Modeling Study in the ABCD Cohort

**Published:** 2026-04-06

**Authors:** Mengman Wei, Qian Peng

**Affiliations:** 1 Department of Neuroscience, The Scripps Research Institute, 10550 N Torrey Pines Rd, La Jolla, 92037, CA, U.S.

**Keywords:** adolescence, ABCD Study, substance use initiation, time-varying Cox model, polygenic risk score, marginal structural model, environmental factors

## Abstract

**Background:**

Early initiation of alcohol, nicotine, cannabis, and other substances is a robust predictor of later substance use disorders and related psychopathology. We integrate time-varying environmental factors with polygenic risk scores (PRS) in a longitudinal framework to identify risk factors influencing substance initiation in adolescents.

**Methods:**

We analyzed data from the Adolescent Brain Cognitive Development (ABCD) Study^®^ with repeated assessments from baseline through approximately four years of follow-up. For each substance (alcohol, nicotine, cannabis, and any substance), we defined a time-to-event outcome indexing age at first use. We assembled a high-dimensional panel of time-varying environmental covariates across multiple domains (family, neighborhood, school, mental health, cognition, and physical health etc.), along with time-invariant covariates and PRS for problematic alcohol use, cannabis use disorder, nicotine use disorder, and any substance use disorder. We constructed individual-level start–stop interval data using interview age in months and fitted time-varying Cox proportional hazards models with robust standard errors clustered by individual ID. First, we conducted univariate models for each predictor. Second, for each outcome, we fitted multivariable Cox models including core covariates (sex, age, site, ancestry principal components), all PRS, and selected predictors. In secondary analyses, we applied marginal structural models with inverse probability of treatment weighting to a subset of modifiable predictors to approximate causal effects under standard assumptions.

**Results:**

In univariate models, earlier initiation was broadly associated with multiple time-varying variables, including impulsivity and externalizing behaviors, sleep disturbance, parenting and monitoring, medication and caffeine use, school functioning and absenteeism, and cultural or value-based measures, alongside other mental health and behavioral factors. In multivariable Cox models, a smaller subset of environmental predictors remained robustly associated with the hazard of initiation across alcohol, nicotine, cannabis, and any substance, highlighting consistent signals in impulsivity traits, parental monitoring, and select health and lifestyle factors. PRS for alcohol use disorder (AUD), cannabis use disorder (CUD), nicotine dependence, and any SUD were positively associated with earlier initiation (hazard ratio [HR] > 1), with the strongest and most consistent signal observed for nicotine PRS (e.g., alcohol initiation HR ≈ 2.37; any-substance initiation HR ≈ 2.98). AUD PRS showed weaker associations for alcohol initiation but stronger associations for any-substance initiation. Causal analyses suggested that parental monitoring (PMQ mean), UPPS lack of planning, UPPS sensation seeking, and caffeine exposure may influence time to initiation: higher parental monitoring was protective (odds ratio [OR] ≈ 0.33–0.64), whereas higher impulsivity traits and caffeine exposure were associated with increased risk (OR ≈ 1.47–3.87) across outcomes, with conclusions robust across weighting specifications.

**Conclusions:**

Integrating time-varying environmental predictors with polygenic risk in a survival framework helps identify environmental factors most strongly associated with earlier substance use initiation beyond genetic liability. Follow-up causal analyses further highlight potentially actionable pathways, particularly parenting and monitoring and impulsivity-related traits, that may contribute to the developmental trajectory leading to adolescent substance use disorders.

## Introduction

Early initiation of alcohol, nicotine, cannabis, and other substances is consistently associated with increased risk of substance use disorders, polysubstance use, and broader externalizing and internalizing psychopathology in later adolescence and adulthood [1, 2, 3, 4]. Understanding which environmental and genetic factors shape the timing of initiation is a critical step toward designing effective preventive interventions.

The Adolescent Brain Cognitive Development (ABCD) Study^®^ [5, 6, 7] provides a unique opportunity to study the development trajectory of these initiations in youth. ABCD follows a large, diverse cohort of U.S. youth with repeated assessments of substance use, demographic and socioeconomic context, family and peer functioning, neighborhood characteristics, mental and physical health, cognition, and neuroimaging, alongside genome-wide genotyping. This allows us to study substance use initiation as a time-to-event outcome under rich longitudinal covariate information.

In this work, we focus on two main aims: Aim 1 (Association): To identify time-varying environmental and polygenic predictors of substance use initiation (alcohol, nicotine, cannabis, and any substance) using time-varying Cox proportional hazards models [8].

Aim 2 (Causal exploration): To perform focused causal analyses for a subset of modifiable environmental predictors that show robust associations in Aim 1, using marginal structural models [9, 10] with inverse probability of treatment weighting.

## Methods

### Study Cohort

We used data from the Adolescent Brain Cognitive Development (ABCD) Study^^®^^ release 5.1 [5]. Participants were included if they had genome-wide genotyping data passing quality control, at least one assessment of substance use, and complete data for core covariates (sex, age, study site, and ancestry principal components). The analytic sample comprised 11,868 children from the ABCD baseline cohort (mean baseline age: 9.91 ± 0.62 years). Longitudinal follow-up data were available for approximately four years.

Substance use initiation was defined separately for alcohol, nicotine, cannabis, and any substance [11]. Time-to-event variables were constructed as the number of months from baseline to first reported use. Participants who did not report initiation were censored at their last available follow-up assessment. By the end of follow-up, initiation events were observed for alcohol (36.5%), nicotine (5.44%), cannabis (3.42%), and any substance (39.7%). Detailed distributions of initiation timing, as well as stratified summaries by sex and race/ethnicity, are provided in the Supplementary Materials.

### Time-varying covariates and candidate predictors

We constructed time-varying environmental covariates from the Adolescent Brain Cognitive Development (ABCD) Study^^®^^ curated core tables. For each participant, we merged all available long-format variables across visits from multiple domains, including demographics and socioeconomic status, culture and environment, gender identity and sexual health, linked external data, mental health, neurocognition, novel technologies, and physical health. For each subject visit, these variables were aligned with the ABCD longitudinal tracking file to obtain interview age, and follow-up time was computed as months since baseline.

As adjustment covariates, we included sex, age at baseline, recruitment site, and the first 20 genetic principal components. These variables were retained in all models and treated as covariates rather than exposures of interest. In addition, we included four polygenic risk scores (PRS) as candidate predictors: problematic alcohol use (AUD) [12], cannabis use disorder (CUD) [13], nicotine-related traits (NUD) [14], and any substance use disorder (anySUD) [15].

We applied minimal filtering to candidate predictors to avoid discarding potentially informative but sparse or baseline-only variables. Specifically, we excluded (i) variables that were entirely missing, (ii) near-constant numeric variables, and (iii) highly collinear numeric variables (absolute pairwise correlation > 0.9). Baseline-only measures were retained and treated as time-varying covariates that remain constant across follow-up.

Polygenic risk scores were computed using external GWAS summary statistics and the PRS-CS [16] and PRS-CSx [17] pipelines. PRS were treated as time-invariant baseline covariates and carried forward across all time intervals for each participant. Detailed procedures for PRS construction are provided in the Supplementary Materials.

### Outcomes: time to substance use initiation

For each ABCD participant, we defined four substance-use initiation outcomes (alcohol, nicotine, cannabis, and any substance) following logic similar to prior work [11]. Substance use was assessed using the Youth Substance Use Interview (annual in-person; su_y_sui.csv) and the Substance Use Phone Interview (mid-year; su_y_mypi.csv).

We harmonized these two data sources and ordered all observations by study visit (baseline, interim phone interviews between annual visits, and annual follow-ups). Follow-up was censored at the 4-year visit (FU4; 4_year_follow_up_y_arm_1), such that only endorsements occurring on or before FU4 were considered initiation events. For each substance, initiation was defined as the first visit with any affirmative endorsement (coded as 1) among prespecified item sets capturing alcohol use (e.g., sip/full drink and TLFB alcohol use), nicotine use (e.g., cigarettes, e-cigarettes, cigars, chew, hookah, pipes, and other nicotine products), and cannabis use (e.g., puff/use, blunts, edibles, concentrates, tinctures, vaping oils). Responses were coerced to valid binary values (0/1), and invalid responses were treated as missing. To avoid misclassification of religious or ceremonial exposure as initiation, alcohol- and nicotine-related endorsements flagged as occurring exclusively in religious contexts were set to missing prior to aggregation. “Any substance” initiation was defined as initiation of alcohol, nicotine, or cannabis, and additionally included a broader set of “other substance” items (e.g., stimulants, opioids, hallucinogens, inhalants, and other drugs) to capture non-alcohol, non-nicotine, and non-cannabis initiation.

For each outcome, we derived (i) a binary case indicator (initiation by FU4), (ii) the timing of first initiation (used to compute time from baseline to first use, in months), and (iii) a censoring time at FU4 for participants without initiation. Controls were defined as participants with no initiation through FU4 and with documented observation at the 3-year follow-up (FU3; 3_year_follow_up_y_arm_1); participants without FU3 observation were coded as missing for case–control phenotypes.

### Time-varying Cox survival models

We analyzed four outcomes (alcohol initiation, cannabis initiation, nicotine initiation, and any substance initiation) using time-varying Cox proportional hazards models [8]. The analysis used three main steps implemented: (1) build a harmonized longitudinal covariate table across study visits, (2) convert each participant’s longitudinal records into start–stop (counting-process) intervals up to their event/censoring time, and (3) fit univariate and multivariable Cox models with robust standard errors.

#### Time scale, outcomes, and overall goal

We studied time until first substance-use initiation (four outcomes: alcohol, cannabis, nicotine, and any substance). For each outcome, every participant had: a follow-up time (the month when they first reported the outcome, or the last month they were observed), and an event indicator (1 = the event happened by that time, 0 = they were censored, meaning we did not observe the event before follow-up ended).

Time was measured in months since the participant’s baseline visit.

Our goal was to link time-varying risk factors (things that can change across visits, like environment or behavior measures) and time-invariant factors (like polygenic risk scores, PRS) to the hazard of initiation (the instantaneous risk of the event at a given time, among those who have not yet initiated).

#### Building the time-varying covariate dataset

Participants were assessed at multiple visits (baseline and follow-ups), with many predictors stored across separate tables. We constructed a unified longitudinal dataset through the following steps:

- **Stacking visits:** For each predictor, we stacked observations across all available visits (baseline and follow-ups) into a long-format structure.
- **Merging predictors:** Predictors from different sources were merged using participant identifiers and visit labels, such that each row corresponded to a single participant at a single visit.
- **Time alignment:** We defined a common time axis using interview age. Interview age was converted to months when necessary, and follow-up time was computed as
time_months=interview_age_months−baseline_age_months,
  so that baseline corresponded to time 0 for all participants.
- **Adjustment variables:** Standard covariates (sex, age, study site, and genetic principal components) were added to all records.
- **Predictor quality control:** We performed basic filtering of predictors by removing (i) variables that were entirely missing, (ii) variables with near-zero variance, and (iii) variables that were highly collinear (i.e., near-duplicate predictors with very high correlation). Sparse predictors (e.g., variables measured only at baseline) were intentionally retained.
- **Integration of PRS:** Polygenic risk scores (PRS) were merged by participant identifier. Although PRS are time-invariant, they were repeated across all visit-level rows for each participant so they could be incorporated alongside other predictors.

#### Construction of Start–Stop (Counting Process) Intervals

Cox models with time-varying predictors require data in interval (“counting process”) form, where each participant contributes multiple rows. Each row represents a time interval [start, stop), predictors are assumed constant within the interval (taking values at the beginning of the interval), and events can occur only at the end of an interval.

For each participant and each outcome, intervals were constructed as follows:

- **Restrict to observed follow-up:** We retained only visits with time_months ≤ the participant-specific follow-up time for the given outcome.
- **Order visits by time:** Visits were sorted in ascending order of time_months.
- **Ensure a common baseline (time 0):** If the first observed visit occurred after month 0, we created a baseline row at time 0 by copying the earliest available observation, ensuring all participants share a common time origin.
- Create consecutive intervals: Let the ordered visit times be $t_{1},t_{2},\ldots ,t_{n}$. Intervals were defined as:
starti={0,i=1,ti,i>1}stopi={ti+1,i<n,T,i=n}
  where T denotes the participant-specific follow-up time. The interval-level event indicator was defined as event_interval = 1 only for the final interval if the participant experienced the event, and 0 otherwise.
- **Remove invalid intervals:** Intervals with stop ≤ start were excluded.

This procedure yields a standard Cox start–stop dataset, in which each participant contributes one or more intervals up to the time of event or censoring.

#### Univariate Time-Varying Cox Models

For each outcome (alcohol, nicotine, cannabis, and any substance initiation), we fitted separate univariate Cox proportional hazards models for each candidate predictor while adjusting for standard covariates.

Let $X_{i}(t)$ denote the value of a given predictor for participant i at time t, which may vary over time. Let $C_{i}$ denote the vector of adjustment covariates, including sex, age at baseline, recruitment site, and the first 20 genetic principal components.

The hazard function for participant i at time t was specified as:

$$h_{i}(t\;|\;X_{i}(t),C_{i})=h_{0}(t)\;\text{exp}\left(\beta X_{i}(t)+\gamma^{T}C_{i}\right),$$

where $h_{0}(t)$ is the baseline hazard function, β is the log hazard ratio associated with the predictor $X_{i}(t)$, and γ is the vector of coefficients for the adjustment covariates. The hazard ratio (HR) for the predictor is given by exp(β).

Models were fitted using the start–stop (counting process) formulation implemented via Surv(start, stop, event_interval). Robust standard errors clustered at the participant level were used to account for within-person correlation arising from repeated intervals.

From each model, we reported the effect estimate and associated uncertainty, including the hazard ratio (HR), 95% confidence interval, and corresponding *p*-value, along with the number of participants and observed events.

#### Multivariable Time-Varying Cox Models with Data-Driven Selection

For each outcome, we fitted a multivariable time-varying Cox proportional hazards model after data-driven predictor selection. The final model used the same start–stop (counting process) interval structure and included (i) forced adjustment covariates and (ii) a subset of selected predictors. Study site was incorporated via stratification, allowing the baseline hazard to vary across sites.

Let $X_{i}(t)$ denote the vector of selected predictors for participant i at time t, and let $Z_{i}$ denote the vector of forced adjustment covariates. The hazard function was specified as:

$$h_{i}(t\;|\;X_{i}(t),Z_{i},{\text{site}}_{i})=h_{0,{\text{site}}_{i}}(t)\;\text{exp}\left(\beta^{T}X_{i}(t)+\gamma^{T}Z_{i}\right),$$

where $h_{0,{\text{site}}_{i}}(t)$ is the site-specific baseline hazard, β are coefficients for selected predictors, and γ are coefficients for adjustment covariates. The forced covariates $Z_{i}$ included sex, age at baseline, and genetic principal components (PC1–PC20). Robust standard errors clustered at the participant level were used to account for within-person correlation.

##### Preventing time leakage

Some predictors were not measured at every visit and may only appear later during follow-up. To avoid incorporating future information, we applied a forward-fill (last observation carried forward) strategy within each participant, such that observed values were propagated to subsequent intervals. No back-filling into earlier time points was performed.

##### Handling missingness

For numeric predictors with intermittent missingness, we adopted a “value + availability” representation. Specifically, for each predictor X, we included (i) an indicator variable $X_{\mathrm{obs}}$ (1 if observed, 0 otherwise), and (ii) an imputed value where missing entries were replaced with the median of observed values. This approach allows the model to distinguish between low values and unavailable measurements, while retaining predictors that begin to be collected later in follow-up.

##### Pre-selection of predictors

To reduce dimensionality and avoid extremely sparse predictors, we applied two filters prior to model selection: (i) a univariate screening step retaining predictors showing evidence of association with the outcome, and (ii) a coverage filter requiring that a predictor be observed at least once in at least 50% of participants. Forced covariates (sex, age, site, and PCs) were always retained for confounding control and were not subject to selection.

##### LASSO-based selection

We applied cross-validated LASSO to select a subset of predictors for each outcome. Because standard LASSO implementations do not fully accommodate start–stop risk sets, LASSO was used as a screening step based on interval end times. Predictors with non-zero coefficients at the selected penalty level were retained for the final model.

##### Final multivariable Cox model

Using the selected predictors, we fitted the final counting-process Cox model with Surv(start, stop, event_interval) as the outcome. The model included selected predictors (and corresponding availability indicators where applicable) and forced adjustment covariates (sex, age, site, and PCs). Robust standard errors clustered by participant were used. We reported hazard ratios (HRs), 95% confidence intervals, *p*-values, and the number of participants and events.

### Causal Inference Analysis: Marginal Structural Models with IPTW

To estimate the causal effects of time-varying exposures on the risk of substance use initiation, we applied marginal structural models (MSMs) with inverse probability of treatment weighting (IPTW) using the start–stop interval dataset. Each participant contributed multiple rows, corresponding to follow-up intervals, with an indicator for whether initiation occurred at the end of each interval. Analyses were conducted separately for alcohol, nicotine, cannabis, and any substance initiation. Baseline covariates included sex, age at baseline, recruitment site, and genetic principal components.

#### Stabilized weights

Let $A_{ik}$ denote the exposure status for participant i at interval k, $Y_{ik}$ the event indicator at the end of the interval, $Z_{i}$ baseline covariates, and $L_{ik}$ time-varying confounders. Stabilized inverse probability weights were defined as:

$$SW_{ik}=\prod_{m=1}^{k}\frac{P(A_{im}\;|\;A_{i,m-1},Z_{i},t_{im})}{P(A_{im}\;|\;A_{i,m-1},Z_{i},t_{im},L_{im})}.$$

Weighted outcome model

The causal effect was estimated using a weighted pooled logistic regression model:

$$\mathrm{logit}\{P(Y_{ik}=1)\}=\alpha (t_{ik})+\psi A_{ik}+\eta^{T}Z_{i},$$

fitted using weights $SW_{ik}$, where α(t) is a flexible function of time and ψ is the causal log-odds ratio associated with exposure.

#### Exposure definition and missingness handling

For each candidate exposure, we defined a binary treatment variable at each interval. Binary variables were used directly, whereas continuous variables were dichotomized using a median split among observed values. To handle missing or late-measured variables without introducing future information, we adopted a “value + availability” representation: an indicator variable (1 = observed, 0 = missing) was included, and missing values were imputed using the median of observed values.

#### Estimation of IPTW

Stabilized weights were estimated using logistic regression models for treatment assignment. The numerator model included prior treatment history, baseline covariates, and flexible functions of time. The denominator model additionally included a selected set of time-varying covariates as potential confounders.

#### Weight diagnostics and stabilization

To mitigate bias due to violations of the positivity assumption and extreme weights, we excluded exposures with very low or very high prevalence, clipped estimated treatment probabilities away from 0 and 1, truncated weights at an upper quantile, and applied predefined weight-quality criteria prior to outcome modeling.

#### Inference

Exposure effects were estimated using weighted pooled logistic regression over intervals, modeling the probability of initiation at each interval end. Participant-level clustered robust standard errors were used to account for repeated measures. For each outcome, only results passing weight-stability and numerical diagnostics were retained. Multiple testing across exposures was controlled using the Benjamini–Hochberg false discovery rate (FDR), and we report odds ratios (ORs), 95% confidence intervals, *p*-values, and FDR-adjusted q-values.

## Results and Discussions

### Study Cohort Characteristics

The analytic sample comprised 11,868 children from the ABCD baseline cohort (mean baseline age: 9.91 ± 0.62 years). By the end of follow-up, substance-use initiation events were observed for alcohol (4,330/11,868; 36.5%), nicotine (646/11,868; 5.44%), cannabis (406/11,868; 3.42%), and any substance (4,706/11,868; 39.7%).

Among participants with events, the mean (SD) time to initiation (months since baseline) was 20.83 (1.82) for alcohol (median 20.75; IQR 19.42–21.83), 22.65 (1.93) for nicotine (median 22.83; IQR 21.33–24.17), 23.30 (1.70) for cannabis (median 23.42; IQR 22.19–24.65), and 20.93 (1.85) for any substance (median 20.92; IQR 19.50–21.92).

Event rates were higher in males than females for alcohol (39.6% vs 36.7%) and any substance (43.1% vs 39.7%), whereas nicotine initiation was similar between sexes (5.29% in males vs 6.02% in females), as was cannabis initiation (3.76% vs 3.51%). Participants with missing sex information showed lower event rates across all outcomes.

By race/ethnicity, alcohol initiation rates were highest among White participants (42.7%, *n* = 6,173), followed by Other (37.3%, *n* = 1,248), Hispanic (32.6%, *n* = 2,410), Asian (33.3%, *n* = 252), and Black participants (20.1%, *n* = 1,784). A similar pattern was observed for any-substance initiation (White 44.8%, Hispanic 36.9%, Black 25.3%, Other 40.9%, Asian 34.1%). Nicotine and cannabis initiation rates were generally lowest among Asian participants (0.79% and 0.40%, respectively). Detailed distribution plots are provided in the Supplementary Materials.

### Univariate Time-Varying Survival Models Results

**Fig. 1.**
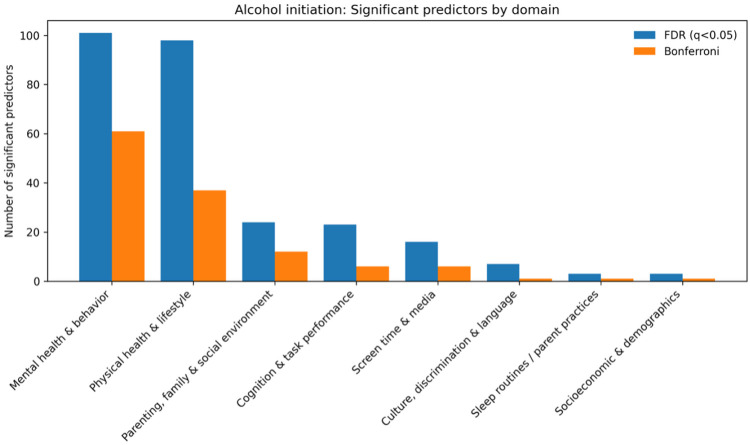
Domain-level counts of significant predictors for alcohol initiation from univariate time-varying Cox models (FDR and Bonferroni thresholds)

Univariate time-varying Cox models identified a broad set of predictors associated with alcohol use initiation after correction for multiple testing (Figure 1). Among 277 predictors that were significant at the false discovery rate (FDR; *q* < 0.05), 125 also met the Bonferroni threshold, indicating robust associations.

Significant predictors clustered primarily within mental health and behavioral domains (accounting for the largest share of Bonferroni-significant hits), followed by physical health and lifestyle factors, and parenting, family, and social environment measures. Overall, these patterns suggest that alcohol initiation risk reflects a combination of behavioral dysregulation, psychosocial stressors, family context and monitoring, and broader health and lifestyle correlates. Social-context measures (e.g., peer- and school-related factors) and discrimination or cultural-context measures were also associated with initiation risk, consistent with the influence of environmental exposures and social experiences.

Because these models were univariate, the findings should be interpreted as correlational and may reflect confounding or shared variance with other behavioral and family factors.

Risk-increasing predictors (hazard ratio [HR] > 1) included measures of youth behavioral dysregulation and adverse social context. For example, youth externalizing problems (BPM externalizing raw: HR = 1.10, 95% CI 1.08–1.12) and involvement with rule-breaking or delinquent peers (PBP rule-break: HR = 1.14, 95% CI 1.11–1.16) were associated with higher hazard (earlier initiation). Discrimination exposure also showed a strong positive association (Discrimination Measure mean: HR = 1.41, 95% CI 1.28–1.55).

In contrast, protective predictors (HR < 1) included greater parental monitoring, which was associated with a lower hazard of initiation (Parental Monitoring mean: HR = 0.82, 95% CI 0.75–0.88). Consistent with a lifestyle and behavioral pathway, several screen and media-related indicators were associated with increased risk, including having a social media account (HR = 1.34, 95% CI 1.20–1.50), more frequent phone checking (HR = 1.05, 95% CI 1.03–1.07), and parent screen use around the child (HR = 1.11, 95% CI 1.06–1.16).

**Fig. 2.**
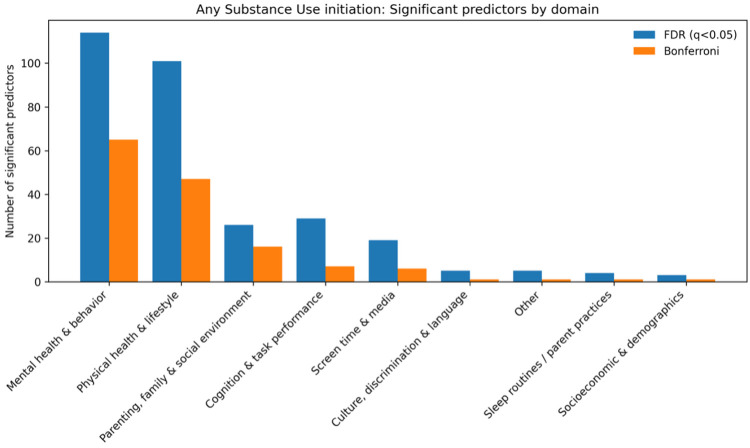
Domain-level counts of significant predictors for any substance initiation from univariate time-varying Cox models (FDR and Bonferroni thresholds)

For any substance use initiation, significant predictors spanned multiple domains, with the largest contributions from mental health and behavior (130 FDR-significant; 75 Bonferroni-significant) and physical health and lifestyle factors (93 FDR; 40 Bonferroni). (Figure 2) Additional signals were observed for parenting, family, and social environment (24 FDR; 22 Bonferroni), cognition and task performance (42 FDR; 15 Bonferroni), sleep routines and parent practices (17 FDR; 11 Bonferroni), and screen time and media use (12 FDR; 8 Bonferroni). Smaller but consistent sets of associations were also identified in other domains (4 FDR; 4 Bonferroni) and in culture, discrimination, and language (2 FDR; 2 Bonferroni).

In terms of direction, risk-increasing factors (hazard ratio [HR] > 1) included higher youth behavioral dysregulation (e.g., demo_SES_long_family_income_cat: HR = 1.07, 95% CI 1.04–1.10) and greater involvement with rule-breaking peers (e.g., dm_long_dim_y_ss_mean: HR = 1.45, 95% CI 1.32–1.58). Protective factors (HR < 1) again included parental monitoring, with stronger monitoring associated with a lower risk of initiation (e.g., crpbi_long_crpbi_y_ss_parent: HR = 0.74, 95% CI 0.68–0.81).

**Fig. 3.**
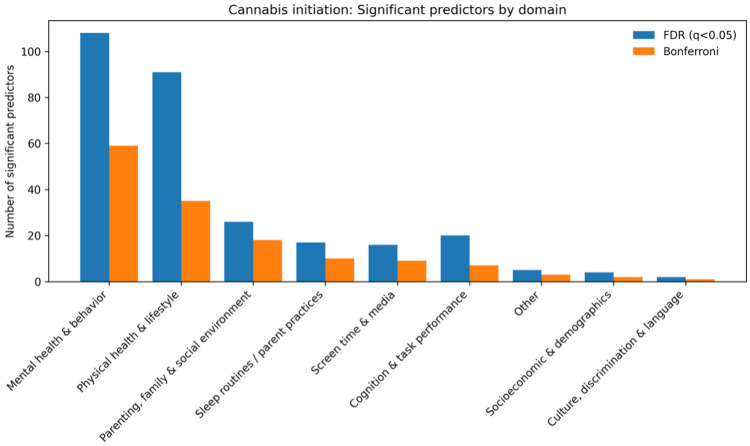
Domain-level counts of significant predictors for nicotine initiation from univariate time-varying Cox models (FDR and Bonferroni thresholds)

For nicotine use initiation, the domain distribution of significant predictors closely mirrored that observed for the “any substance” outcome. The largest contributions arose from mental health and behavior (130 FDR-significant; 75 Bonferroni-significant) and physical health and lifestyle factors (93 FDR; 40 Bonferroni). Additional signals were identified for parenting, family, and social environment (24 FDR; 22 Bonferroni), cognition and task performance (42 FDR; 15 Bonferroni), sleep routines and parent practices (17 FDR; 11 Bonferroni), screen time and media use (12 FDR; 8 Bonferroni), other domains (4 FDR; 4 Bonferroni), and culture, discrimination, and language (2 FDR; 2 Bonferroni).(Figure 3)

Risk-increasing factors (hazard ratio [HR] > 1) included higher youth behavioral dysregulation (e.g., comc_long_comc_ss_control_p: HR = 1.01, 95% CI 1.00–1.01) and greater involvement with rule-breaking peers (e.g., dm_long_dim_y_ss_mean: HR = 1.96, 95% CI 1.64–2.35). Protective factors (HR < 1) again included parental monitoring, with stronger monitoring associated with a lower hazard of nicotine initiation (e.g., crpbi_long_crpbi_y_ss_parent: HR = 0.39, 95% CI 0.32–0.47).

**Fig. 4.**
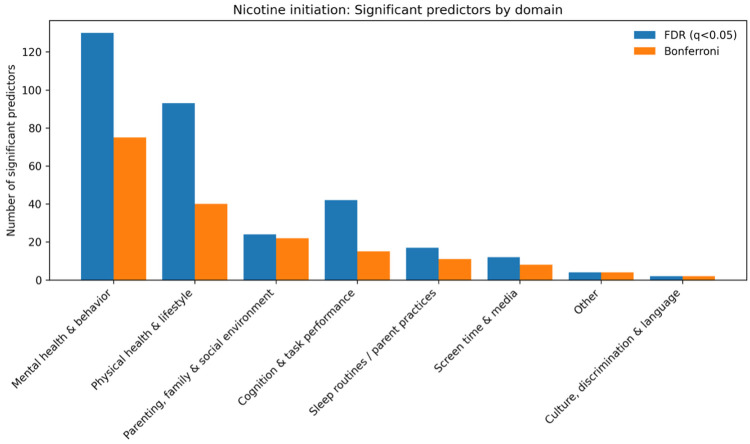
Domain-level counts of significant predictors for cannabis initiation from univariate time-varying Cox models (FDR and Bonferroni thresholds)

For cannabis use initiation, significant predictors were again distributed across multiple domains, led by mental health and behavior (75 FDR; 40 Bonferroni) and physical health and lifestyle (40 FDR; 22 Bonferroni). Additional contributions were observed for parenting, family, and social environment (22 FDR; 14 Bonferroni), cognition and task performance (15 FDR; 7 Bonferroni), sleep routines and parent practices (11 FDR; 5 Bonferroni), screen time and media use (8 FDR; 4 Bonferroni), and other domains (4 FDR; 4 Bonferroni).(Figure 4)

Risk-increasing factors (HR > 1) included higher youth behavioral dysregulation (e.g., dm_long_dim_y_ss_mean: HR = 1.89, 95% CI 1.52–2.36) and peer rule-breaking involvement (e.g., pbp_long_pbp_ss_rule_break: HR = 1.48, 95% CI 1.41–1.54). Protective factors (HR < 1) included parental monitoring and broader family context measures; for example, parent married or cohabiting status was associated with a lower hazard of cannabis initiation (e.g., demo_SES_long_parent_married_or_cohab: HR = 0.51, 95% CI 0.39–0.67).

Across traits, a similar overall pattern emerged. Mental health and behavioral domains consistently contributed the largest number of significant predictors across all substances. Physical health and lifestyle factors, as well as parenting/social environment measures, also made substantial contributions, particularly for protective associations related to parental monitoring and socioeconomic or family context. In addition, screen time/media indicators and peer rule-breaking involvement showed consistent risk-increasing associations with earlier initiation across outcomes.

Taken together, these results suggest that substance use initiation is strongly linked to behavioral and psychosocial factors (including mental health symptoms, peer involvement, and family/social environment), while lifestyle and physical health factors (such as screen use patterns and related behaviors) provide additional, meaningful predictive signal. Social-context measures (including discrimination, peer relationships, and parental practices) also appear relevant and may help explain initiation risk. However, because these analyses used univariate Cox models, the observed associations should be interpreted cautiously, as they may reflect confounding and shared variance among correlated predictors.

#### Polygenic risk scores (PRS) and initiation risk

In univariate survival models, higher polygenic risk was broadly associated with increased hazard of substance use initiation across alcohol, nicotine, cannabis, and any substance outcomes. The most consistent evidence was observed for cannabis and nicotine initiation, for which multiple PRSs showed positive associations. For alcohol initiation, associations were more selective, with some PRSs showing clearer evidence than others. Although several associations were statistically supported, some effect estimates were accompanied by wide confidence intervals, particularly for cannabis and nicotine outcomes, indicating limited precision in their magnitude. Taken together, these results suggest that elevated polygenic liability to substance-related traits is generally associated with earlier initiation. Full numerical results are presented in the Supplementary Materials.

### Multivariable Time-Varying Survival Models Results

#### Cross-outcome signals and overall patterns

##### Multivariable model results.

Across multivariable models, the most consistent cross-outcome signal was peer delinquency (rule-breaking), which was associated with a higher hazard of initiation for all four outcomes, with the largest effect sizes observed for cannabis and nicotine:

- **Alcohol**: HR = 1.052 (95% CI 1.029–1.075), *p*_Bonf_ = 8.35 × 10^−4^
- **Any substance:** HR = 1.058 (95% CI 1.037–1.080), *p*_Bonf_ = 7.98 × 10^−6^
- **Cannabis:** HR = 1.292 (95% CI 1.233–1.354), *p*_Bonf_ = 2.64 × 10^−25^
- **Nicotine:** HR = 1.168 (95% CI 1.120–1.218), *p*_Bonf_ = 3.03 × 10^−11^

Beyond this shared signal, outcomes separated into two broad profiles. Alcohol and any-substance initiation exhibited a broader psychosocial signature spanning impulsivity, peer context, family environment, and additional domains including stress, sleep, and sociodemographic and cultural context. In contrast, cannabis and nicotine initiation showed a more tightly clustered profile centered on externalizing and rule-breaking behavior, characterized by the combination of peer delinquency and parent-reported CBCL rule-breaking. Cannabis initiation had the fewest observed events and correspondingly fewer multivariable signals; however, the identified predictors were strong and internally coherent.

#### Trait-Specific Multivariable Results

##### Alcohol initiation

In the alcohol model, six predictors remained Bonferroni-significant, and all were associated with increased hazard. These included an impulsivity-related signal (youth sensation seeking), the shared cross-outcome peer effect (delinquent peers), and family environment (family conflict), along with distress-related and additional correlates:

- ders_parent_long_ders_attuned_nm (Parent DERS attuned missing-items count): HR = 1.259 (95% CI 1.190–1.331), *p* = 1.23 × 10^−13^
  *Note: likely reflects missingness or assessment-quality signal; interpret cautiously*.
- upps_y...sensation_seeking (Child sensation seeking): HR = 1.118 (95% CI 1.075–1.163), *p* = 4.29 × 10^−6^
- pps_y...bother_sum_obs (Bother/distress summary, observed/raw) HR = 1.299 (95% CI 1.182–1.426), *p* = 1.06 × 10^−5^
- pbp...rule_break (Delinquent peers): HR = 1.052 (95% CI 1.029–1.075), *p* = 8.35 × 10^−4^
- fes...fc_pr (Family conflict): HR = 1.075 (95% CI 1.035–1.117), *p* = 0.0010
- bfq...breastfeed_p (Breastfed history): HR = 1.186 (95% CI 1.082–1.300), *p* = 0.0042

An additional set of predictors reached significance at the FDR level only, expanding the alcohol risk profile to include sociodemographic, cultural, stress, and sleep-related correlates. These included cyberbullying perpetration (cbb...harm2: HR = 1.448, *q* = 0.0123), family income category (HR = 1.071, *q* = 0.0482), household size (protective; HR = 0.652, *q* = 0.0482), Mexican American cultural values (protective; HR = 0.931, *q* = 0.0482), perceived stress (unexpected upset; HR = 1.034, *q* = 0.0482), and a sleep disturbance item (vivid dream-like scenes; HR = 1.061, *q* = 0.0482).

Overall, multivariable results indicate that alcohol initiation is most strongly associated with a combination of impulsivity-related traits, peer delinquency, and family conflict, with additional contributions from stress, sleep disturbance, and sociodemographic or cultural factors. Notably, the most consistent signals reflect behavioral dysregulation and adverse social context, whereas measurement-related predictors (e.g., missingness counts and _obs fields) should be interpreted cautiously as potential correlates of reporting structure or participant engagement.

##### Any substance initiation

The any-substance model identified six Bonferroni-significant predictors. The strongest signals again reflected peer context and behavioral dysregulation, alongside variables whose interpretation depends on measurement properties or scaling:

- ders...attuned_nm (Parent DERS attuned missing-items count): HR = 2.901 (95% CI 2.214–3.801), *p* = 1.92 × 10^−10^
  *Note: likely reflects assessment or missingness artifact; interpret cautiously*.
- ddis...mnrt_immcho (Delay discounting reaction time): HR ≈ 1.000 per 1 ms, *p* = 3.84 × 10^−10^
  *Note: statistically significant but very small per-unit effect; rescaling improves interpretability*.
- Bother/distress (observed/raw): HR = 1.314 (95% CI 1.198–1.440), *p* = 1.06 × 10^−5^
- Delinquent peers: HR = 1.058 (95% CI 1.037–1.080), *p* = 7.98 × 10^−6^
- Sensation seeking: HR = 1.033 (95% CI 1.019–1.047), *p* = 0.0014
- Breastfed history: HR = 1.184 (95% CI 1.087–1.290), *p* = 0.0015

Additional predictors reached significance at the FDR level only, highlighting contributions from sleep and family environment domains. These included a sleep-disordered breathing item (protective; HR = 0.753, *q* = 0.0258), prorated family environment expressiveness and conflict (HR = 1.037, *q* = 0.0365; HR = 1.027, *q* = 0.0429), parent CBCL rule-breaking (HR = 1.028, *q* = 0.0429), CBD permission (HR = 1.309, *q* = 0.0457), and vivid dream-related sleep disturbance (HR = 1.101, *q* = 0.0429).

Overall, any-substance initiation exhibited a multivariable profile similar to alcohol, characterized by peer delinquency, impulsivity or behavioral dysregulation, and family environment, with additional contributions from sleep-related factors. Notably, several highly significant predictors reflect metadata or fine-grained measurement scales (e.g., missingness counts and millisecond-level reaction time), suggesting that some effect sizes may reflect properties of measurement or reporting rather than direct causal effects.

##### Nicotine initiation

For nicotine initiation, five predictors were Bonferroni-significant, led by peer context and parent-reported rule-breaking, with additional contributions from internalizing-related measures and puberty or metadata indicators:

- Delinquent peers: HR = 1.168 (95% CI 1.120–1.218), *p* = 3.03 × 10^−11^
- Parent CBCL rule-breaking: HR = 1.099 (95% CI 1.063–1.137), *p* = 2.80 × 10^−6^
- Parent CBCL internalizing (protective): HR = 0.961 (95% CI 0.946–0.976), *p* = 4.78 × 10^−5^
- Parent ASR internalizing _obs field (protective): HR = 0.118 (95% CI 0.041–0.335), *p* = 0.0058
  *Note: the large effect size likely reflects measurement availability rather than symptom severity*.
- Puberty scale (male missing-answers count): HR = 1.383 (95% CI 1.160–1.648), *p* = 0.0296

Three additional predictors reached significance at the FDR level only, including a depressed mood item (bpm...unhappy_sad_depressed: HR = 1.306, *q* = 0.0176), relative social jetlag (HR = 1.068, *q* = 0.0491), and puberty-related skin changes (pimples; HR = 1.202, *q* = 0.0494).

Overall, nicotine initiation was most strongly associated with externalizing social context (peer delinquency) and parent-reported rule-breaking, with additional contributions from mood symptoms, circadian misalignment (social jetlag), and puberty-related measures. Two internalizing-related parent-report measures appeared protective; however, the very large effect estimate for the ASR internalizing _obs field likely reflects measurement availability rather than symptom severity and should be interpreted cautiously.

##### Cannabis initiation

Cannabis initiation exhibited a compact multivariable signature, with three Bonferroni-significant predictors:

- Delinquent peers: HR = 1.292 (95% CI 1.233–1.354), *p* = 2.64 × 10^−25^
- Parent CBCL rule-breaking: HR = 1.136 (95% CI 1.097–1.176), *p* = 8.10 × 10^−4^
- Neighborhood collective capacity (comc...collective_capacity_p): HR = 1.005 (95% CI 1.003–1.007), *p* = 0.0030

Two additional predictors reached significance at the FDR level only: regular school attendance (protective; HR = 0.477, *q* = 0.0157) and CBD permission (HR = 1.939, *q* = 0.0437).

Despite fewer observed events (and correspondingly fewer retained predictors), cannabis initiation was strongly and consistently associated with an externalizing and rule-breaking profile, led by peer delinquency and parent-reported rule-breaking. Regular school attendance emerged as a protective factor. Neighborhood context also contributed; however, the modest per-unit effect size highlights the importance of scale when interpreting continuous community-level measures.

### Causal Analyses Results

Using marginal structural models with inverse probability of treatment weighting (MSM–IPTW) and pooled logistic regression over intervalized follow-up, we identified a concentrated set of weight-stable causal candidates. Across outcomes, 14–15 exposures met predefined weight-stability criteria, of which 5–6 per outcome were significant after false discovery rate (FDR) correction (*q* ≤ 0.05).

Four exposures were consistently significant across all outcomes: past 24-hour caffeine exposure (risk-increasing), lower parental monitoring (protective), and two impulsivity-related traits (lack of planning and sensation seeking; risk-increasing).

Additional outcome-specific associations were observed. A culture and values measure showed a protective effect for alcohol, any substance, and cannabis initiation. Sleep disturbance measures were associated with increased risk for any substance and cannabis initiation (with lower prevalence for cannabis). For nicotine initiation, skipping school days emerged as a borderline association, likely reflecting limited prevalence.

**Fig. 5.**
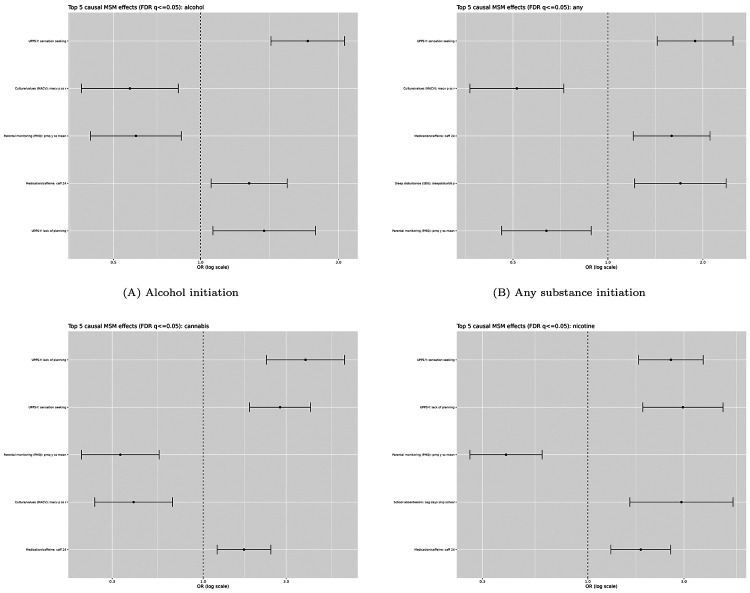
Top causal effects estimated using MSM–IPTW models across substance use initiation outcomes (FDR *q* < 0.05). Points represent estimated odds ratios with 95% confidence intervals. Panel (A): alcohol initiation; panel (B): any substance initiation; panel (C): cannabis initiation; panel (D): nicotine initiation.

These findings were robust to alternative propensity score clipping and truncation strategies, with stable weight diagnostics across sensitivity analyses (Figures 5).

## Conclusion

In this large longitudinal study, time-varying Cox models identified multiple environmental domains that were associated with earlier initiation of alcohol, nicotine, cannabis, and any substance, indicating that children’s environments contribute to initiation risk beyond genetic predisposition. In parallel, polygenic risk scores for substance-related traits were consistently associated with earlier initiation, supporting the idea that genetic liability influences not only problematic use outcomes but also the timing of first onset.

Across outcomes, the most consistent signals pointed to a core set of psychosocial domains, particularly peer context and family functioning. Peer delinquency and rule-breaking emerged as a robust cross-outcome correlate, while family environment and parenting-related measures frequently appeared as protective or risk-related factors.

The time-varying framework is well-suited to capturing shifting risk dynamics during development, when exposures such as peer networks, family monitoring, stress, sleep, and school engagement can change rapidly. This dynamic perspective has practical implications: it suggests that “when” exposures change may matter nearly as much as “which” exposures matter, and that prevention strategies may be more effective when they are timed to periods of heightened vulnerability rather than relying on baseline-only risk profiles.

The PRS results complement the environmental findings by helping explain why some youth may be more vulnerable to earlier initiation across substances. At the same time, these results should not be interpreted as supporting PRS-based screening in clinical or school settings at this stage, as current PRSs explain a limited proportion of variance.

This study has several strengths. The analysis leverages a large, diverse longitudinal sample with repeated assessments across many environmental domains, enabling systematic evaluation of time-varying predictors. Integrating polygenic risk with longitudinal environmental measures provides a more complete picture of initiation risk than either component alone. Methodologically, the time-varying survival framework, combined with robust standard errors clustered by individual, helps address repeated intervals and supports more realistic modeling of developmental change. The additional step toward causal modeling using marginal structural models helps further understand these correlations.

Several limitations should also be emphasized. Many predictors are derived from multi-item instruments and can be affected by measurement error, reporting bias, or instrument-specific scaling, which may attenuate effect estimates. Although the models adjust for a broad set of covariates, residual confounding remains possible, especially for causal analyses where unmeasured factors may influence both exposure trajectories and initiation risk.

Future work can build directly on these findings by testing effect modification by polygenic risk (gene–environment interaction) to identify whether certain environmental factors have stronger associations among genetically higher-risk youth. Incorporating neuroimaging measures as potential intermediate phenotypes could help link genetic liability and environmental exposure to neurodevelopmental pathways that precede initiation.

In summary, using time-varying survival models alongside polygenic risk scores, we mapped environmental and genetic predictors of substance use initiation in the ABCD cohort. The results highlight a set of modifiable environmental factors that remain associated with initiation after accounting for genetic liability, while PRS contributes additional evidence for underlying vulnerability to earlier onset. Follow-up causal modeling suggests that sustained changes in a subset of exposures may meaningfully delay initiation, supporting their potential value as targets for prevention and early intervention efforts.

## Data Availability

The analysis code and scripts used in this study are freely available at the following GitHub repository: https://github.com/mw742/ABCD_sur_cau. This study uses data from the Adolescent Brain Cognitive Development (ABCD) Study (https://abcdstudy.org), held in the NIMH Data Archive (NDA). The ABCD data release used was version 5.1. The study is supported by the National Institutes of Health (NIH) and additional federal partners under multiple award numbers, including U01DA041048 and U01DA050987. The full list of funders is available at https://abcdstudy.org/federal-partners.html.
